# Severe Thrombotic Thrombocytopenic Purpura (TTP) with Organ Failure in Critically Ill Patients

**DOI:** 10.3390/jcm11041103

**Published:** 2022-02-19

**Authors:** Sofiane Fodil, Lara Zafrani

**Affiliations:** Medical Intensive Care Unit, Saint-Louis Hospital, Assistance Publique des Hôpitaux de Paris, University of Paris, 75010 Paris, France; lara.zafrani@aphp.fr

**Keywords:** thrombotic thrombocytopenic purpura, intensive care unit, organ failure

## Abstract

Thrombotic thrombocytopenic purpura (TTP) is a multiorgan disorder. Organ dysfunction occurs as a consequence of widespread microvascular thrombosis, especially in the heart, brain and kidney, causing transient or partial occlusion of vessels, resulting in organ ischemia. Intensive care unit (ICU) admission varies between 40% and 100% of patients with TTP, either because of severe organ failure or in order to initiate emergency plasma exchange (PEx). Severe neurologic manifestations and cardiac involvement have been associated with higher mortality. Acute kidney injury, although usually less severe than that in hemolytic and uremic syndrome, is common during TTP. Initial management in the ICU should always be considered in TTP patients. The current treatment of TTP in the acute phase is based on urgent PEx, combined with corticosteroid therapy, B-cell-targeted immunotherapy, rituximab and inhibition of the interaction between ultra-large Von Willebrand factor multimers and platelets, using caplacizumab, a monoclonal antibody. ICU management permits close monitoring and the rapid introduction of life-sustaining therapies. This review details the epidemiology of TTP in the ICU, organ failures of critically ill patients with TTP, and the initial management of TTP patients in the ICU.

## 1. Introduction

Thrombotic thrombocytopenic purpura (TTP) is a thrombotic microangiopathy (TMA), characterized by spontaneous thrombus formation in the microcirculation. The diagnosis of TMA relies on the combination of mechanical hemolytic anemia (which results in the production of fragmented red blood cells (schizocytes)), peripheral thrombocytopenia and various signs of organ ischemia due to microvessel thrombosis [1].

TTP is due to a deficiency in the von Willebrand factor (VWF)-cleaving specific metalloprotease ADAMTS13. Under physiological conditions, ADAMTS13 adopts a closed latent conformation and VWF, secreted by platelets and endothelial cells, is in a spherical state. The proteolytic activity of ADAMTS13 on VWF depends on the conformational change in the two proteins.

Under high shear stress, VWF untangles and exposes its A1 domain, which enables interaction with platelets via the GpIb/IX/V complex. The A2 domain of VWF is then elongated and exposes the ADAMTS13 binding sites. The interaction of ADAMTS13 with VWF induces an open conformation of ADAMTS13. In the case of ADAMTS13 deficiency, VWF multimers accumulate, leading to increased platelet adhesion and aggregation, resulting in disseminated microthrombi and organ ischemia. Congenital TTP (cTTP), secondary to recessively inherited mutations of the ADAMTS13 gene, is classically observed in about 5% of all TTP patients [2,3]. Mariotte et al. showed that pregnancy-associated TTP is characterized by a rate of cTTP that is 14 times higher than that observed globally in adult-onset disease [2,4]. Among patients with acquired TTP (aTTP), anti-ADAMTS13 antibodies are identified in 75% of cases (immune-mediated TTP) [1,2]. 

The annual incidence is 1.5–6 cases per million per year in adults and the prevalence of TTP is 10–15 cases per million, with a female:male ratio of 2:1, and a peak incidence of the disease before the age of 50 [2]. It has been suggested that the prevalence of TTP in individuals of blood group O may be lower than in those from other blood groups because of the lower levels of VWF in patients with blood group O [5,6,7]. 

The initial management focuses on eliminating other causes of TMA, hemolytic and uremic syndrome (HUS) and secondary TMA syndromes [8]. HUS encompasses different disorders, including typical HUS, which is due to an infection from shiga toxin-producing *Escherichia coli* (HUS-STEC), as compared with atypical HUS, during which genetic or acquired dysregulation of the complement alternative pathway is detected in half of the patients. HUS is characterized by the presence of endothelial cell injury in the microvasculature of the kidney and, less frequently, in other organs [9].

The diagnosis of TTP is based on clinical and laboratory findings. Once TMA diagnosis is established, an ADAMTS13 activity below 10% determines the diagnosis of TTP, with or without anti-ADAMTS13 antibody detection. TTP is a multiorgan disorder. Organ dysfunction occurs as a result of widespread microvascular thrombosis, especially in the heart, brain and kidney, causing transient or partial occlusion of vessels which results in organ ischemia [10].

In this review, we will detail the epidemiology of TTP in the intensive care unit (ICU), organ failures of critically ill patients with TTP, and the initial management of TTP patients in the ICU.

## 2. Epidemiology of TTP in ICU

The severity of TTP varies, and there is no consensual definition of severe TTP. Nonetheless, any TTP with organ failure should be closely monitored and prompt ICU admission should be arranged. Some expert recommendations even suggest that all patients with suspected TTP should be admitted to the ICU for emergency diagnostic, therapeutic and supportive management. Considering the rare incidence of TTP, experts recommend that patients should preferably be admitted to a referral center with a multidisciplinary team experienced in the management of TTP in intensive and long-term care that includes, at the least, a TTP specialist, a hematologist and an intensivist [11,12]. Various studies show that ICU admission varies between 40% and 100% of patients with TTP, either because of severe organ failure or in order to initiate emergency plasma exchange (PEx), 7 days a week, 24 h a day [13,14]. In ICUs, data on the use of life-sustaining therapy are very scarce. In 2021, Van de Louw et al. reported a large retrospective multicentric cohort of “severe” TTP, with at least one organ failure. In a population of 1096 patients with TTP admitted to the ICU, 17% required one life-sustaining therapy, and 3% required two or more life-preserving therapies, respectively. In total, 9% required mechanical ventilation, 16% required renal replacement therapy (RRT) and 0.6% needed vasopressor therapy [14]. Morbid obesity has been suggested as a risk factor for severe TTP, with an increased risk of cardiovascular and renal complications [15,16]. Before the introduction of caplacizumab, the death rate of TTP patients, treated with PEx, corticosteroids and rituximab, ranged from 15% to 20% in the month following diagnosis [17,18]. Various severity scores have been proposed in TTP patients. Benhamou et al. found that a score that included age, an elevated lactate dehydrogenase (LDH) level higher than 10-fold of the normal, and cerebral involvement, was highly accurate in predicting the risk of death in TTP patients [17]. Goel and colleagues reported the results of a different mortality score in TTP patients that included cerebral involvement, a history of platelet transfusion, acute kidney injury (AKI), myocardial infarction, macrovascular arterial thrombosis and age > 60 years [19]. Some authors have also suggested a grading system for aTTP [20] or cTTP [21] patients, dividing them into five categories based on the severity of clinical symptoms and biological abnormalities encountered during an episode of TTP. Finally, high anti-ADAMTS13 antibody levels and low ADAMTS13 antigen levels have been correlated with mortality [22].

## 3. Organ Injuries in TTP 

### 3.1. Neurological Manifestations in TTP

In the first description of TTP, *Moschcowitz* published the case of a patient with typical TTP and hemiparesis. Pathology analysis revealed hyaline thrombosis of the terminal arterioles and capillaries [23,24]. Further autopsy studies have revealed platelet-rich microvascular thrombi associated with the high expression of VWF in the cerebral cortex or the kidney. These are opposed to fibrin/red cell-rich thrombi in patients with HUS [25,26,27]. Since then, it has been found that neurologic manifestations are predominant in TTP, as they may be encountered in up to 70–90% of patients [28,29,30]. In a cohort of patients with TMA, an ADAMTS13 activity <10% was statistically associated with higher neurological impairment [31]. Neurological symptoms are often temporary and can range from headaches to focal signs, stroke, seizures, or coma. In a retrospective monocentric American study, severe neurological symptoms, defined as focal deficit, a history of seizure and impaired consciousness, have been associated with early mortality [32] and in a retrospective multicenter French study, cerebral manifestations were associated with early death [17]. In TTP patients, reported brain Magnetic Resonance Imaging (MRI) findings are consistent with posterior reversible encephalopathy syndrome (PRES) and ischemic lesions, while hemorrhagic lesions are uncommon [33]. In addition to the specific treatment of TTP, the management of neurological complications includes mechanical ventilation in comatose patients or those suffering from status epilepticus. The most recently published (2021) multicenter study of patients with TTP, ICU admitted or otherwise, shows that, out of 1096 patients, only 314 (28.6%) had neurological signs. In this large study, the presence of neurological signs was not associated with mortality [14]. Mirouse and colleagues have published findings from the largest retrospective cohort of neurological manifestations in patients with TTP admitted to the ICU, from which, 108 out of 130 (83%) patients with TTP exhibited neurological symptoms over a 12-year period [30]. These consisted mainly of headaches in half of the patients, pyramidal syndrome or limb paresis in 32 (30%) and 30 (28%) patients, and language impairment in 23 (22%) subjects. Other manifestations included delirium, epileptic seizures and visual blur. They identified three clusters of patients according to their symptoms. Patients from cluster 1 were younger than patients from clusters 2 and 3, presented more often with headaches and never displayed a decreased level of consciousness. Patients from cluster 2 presented more cases of cerebellar syndrome and more dizziness compared with clusters 1 and 3. Patients from cluster 3 presented more often with delirium and seizures in comparison with patients from clusters 1 and 2, and more frequently demonstrated a decreased level of consciousness. These groups are associated with the longer-term prognosis of patients with no difference regarding cerebral imaging (CT scan or MRI) across the three clusters. Using the Glasgow Outcome Scale (GOS), patients in group 1 had a better recovery at 3 months, 6 months and 1 year than patients in group 2, who had a better recovery than patients in group 3. Improvement in TTP management led to decreased mortality and relapse rates [13,34,35,36]. Among TTP survivors, long-term follow-up revealed significant morbidity with a higher prevalence of cognitive impairment, particularly in tests of visual learning, memory, and depression [37,38,39,40,41,42].

**Figure 1:**
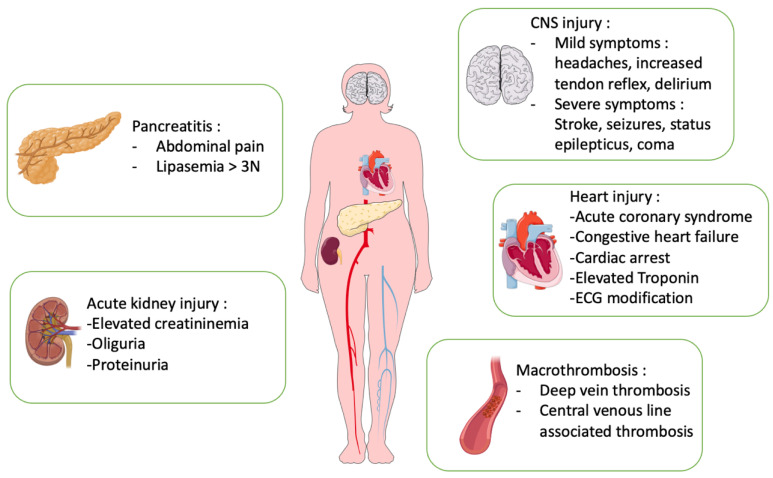
Organ injury in thrombotic thrombocytopenic purpura (TTP). The main organ involvement in TTP is in the central nervous system (CNS), heart, kidneys, pancreas and macroscopic thrombosis.

Patients most at risk may benefit from close monitoring and the treatment of secondary brain damage, as well as early neurological rehabilitation. Further studies on the long-term risk of patients treated with caplacizumab are needed (Figure 1).

### 3.2. Cardiac Manifestations in TTP

Cardiac lesions in TTP are consequences of microthrombi in the small vessels of the coronary arterial network with few associated hemorrhages, described on autopsies. Lesions are mainly localized in the atrioventricular node and His bundle parts [43]. The thrombi are also platelet-rich with a higher density than in the brain. Although some cases have been described as acute myocardial infarction, it is likely that the cardiac lesions range from necrosis in limited areas of microvascular occlusion to extensive trans-mural damage [25,43]. The very first reported case by *Moschcowitz* recognized cardiac involvement in a TTP patient developing heart failure with pulmonary edema [23,24]. Since then, several studies have shown that cardiac involvement in TTP can vary from chest pain or heart failure symptoms associated with electrocardiogram (ECG) changes, elevation of cardiac enzymes, imaging evidence of massive myocardial infarction, cardiomyopathy, arrhythmia, or even sudden death. Performing a systematic review of the literature, *Hawkins* et al. revealed that 24 patients out of a cohort of 111 patients had cardiac symptoms. The most frequent cardiac symptoms were chest pain (*n* = 13, 11.7%), congestive heart failure (*n* = 10, 9.0%), and syncope (*n* = 1, 0.9%). A total of 67 cardiac events were reported, comprising myocardial infarction (*n* = 26, 38.8%), congestive heart failure (*n* = 17, 25.3%), arrhythmias (*n* = 10, 14.9%), cardiogenic shock (*n* = 6, 8.9%) and sudden death (*n* = 8, 11.9%) [44]. In additions to the clinical manifestation of cardiac involvement, it has been established that asymptomatic elevation of cardiac enzymes is more frequent. In 2009, in a small retrospective study, Hughes et al. demonstrated a correlation between death, an elevated cardiac troponin and high levels of anti-ADAMTS 13 IgG antibodies [45]. Benhamou and team reported an elevated cardiac troponin-I in 78 (59%) patients in a cohort of 133 patients. Moreover, they found that an elevated cardiac troponin-I was associated with a higher mortality rate [46]. In a population of TTP patients admitted to the ICU, it was also shown that cardiac involvement, including an isolated elevated troponin level, was associated with TTP unresponsiveness [47]. Later, focusing on 98 critically ill patients with “severe TTP”, Fourmont et al. found that cardiac involvement was present in 89 (91%) patients. The most common cardiac event was an elevated troponin level (*n* = 71, 72%) and elevated troponin was the only sign in 20 cases (20%). ECG alterations were described in 58 (59%) patients, chest pain in 23 (5%) patients, cardiogenic shock in 17 (3%) patients and cardiac arrest in 6 (1%) patients. Transthoracic echocardiography was available in 56 patients and revealed left ventricular dysfunction in 9 patients (16.1%), focal hypokinesia in 11 patients (19.6%) and pericardial effusion in 12 (21.4%) [48]. In 2021, Van de Louw et al. reported a prevalence of 0.6% (*n* = 7) for cardiogenic shock and 5% (*n* = 56) for myocardial infarction in a cohort of 1096 patients [14]. The presence of microvascular cardiac damage, frequent in TTP, and its association in various studies with higher mortality, has raised the question of adjuvant therapy such as anti-platelet aggregation treatment. An early Italian study showed that the addition of an anti-platelet drug (ticlopidine) to PEx reduced mortality at day 15 [49]. Expert recommendations suggest full cardiac work-up upon ICU admission with clinical examination, ECG, echocardiography and troponin assessment. In addition, aspirin is recommended in patients with cardiac involvement once the platelet count is above 50 G/L [11]. In the case of typical acute coronary syndrome, coronary angiography should at least be discussed. A significant number of patients with TTP and acute coronary syndrome have a detectable thrombus on coronary angiography [50,51,52,53,54]. Angioplasty must then be discussed in the case of coronary involvement (Figure 1).

### 3.3. Acute Kidney Injury (AKI) in TTP

Data about renal injury in patients with TTP are scarce. In contrast to renal abnormalities in HUS [9], the clinical, biological and pathophysiological aspects of renal injury in TTP have been poorly characterized. Renal involvement is considered an unusual feature of TTP; however, several renal events can occur in TTP patients. The pathophysiology of AKI in TPP, besides microthrombi in the microcirculation of kidneys [25], encompasses hemolysis, hemodynamic instability, drug-induced renal toxicity or the renal involvement of an underlying connective tissue disease. Prior to the generalization of the ADAMTS13 assessment, the distinction between TTP and HUS was based primarily on the predominance of renal or neurological dysfunction. However, Veyradier et al. showed that some patients with TMA that physicians considered as “presumed HUS” had severe deficiency of ADAMTS13 and therefore had authentic TTP [55]. More recently, several studies, classifying TTP according to ADAMTS13 activity <10%, have demonstrated that AKI in TTP is more frequent than suspected. The study performed by *Van de Louw* and colleagues on 1096 patients found that AKI was observed in 41% of the patients, and 16% of the patients required RRT. In this study, RRT was not associated with mortality. Zafrani et al. showed that in 92 patients admitted to the ICU, there was a prevalence of AKI of 58.7% (*n* = 54), of which 46% (*n* = 25) had stage 3 AKI, according to KDIGO 2012 guidelines [56].

In this cohort, RRT was used in 14 patients (25.9%). The only factor associated with the occurrence of AKI was decreased C3 levels, suggesting an activation of the alternative complement pathway in TTP-induced AKI patients. They also studied the longer-term renal prognosis of these patients and found that only three patients still required chronic dialysis at 6 months after remission of TTP, but mild or moderate chronic renal disease occurred in 23 patients(42.6%) [57]. Nevertheless, AKI is most often less severe than the severity of AKI observed during HUS [58]. Another interesting feature of AKI in TTP is its higher prevalence in patients with cTTP compared with patients with aTTP. One possible explanation has been suggested by Tsai et al., who hypothesized that, in contrast to patients with cTTP, patients with aTTP retain significant local ADAMTS13 production in the kidney that may in part protect these patients from glomerular microangiopathy [21,59]. 

Upon admission to the ICU, serum creatinine measurement is part of the standard work-up. Studies of renal impairment in TTP were conducted prior to the use of caplacizumab. The trials that have registered caplacizumab do not report rates of renal failure or RRT [13,34]. Further studies are needed to assess the prevalence and impact of renal failure in patients with TTP (Figure 1).

### 3.4. Pancreatitis and Gastrointestinal Disorder in TTP

The largest autopsy series of patients with TMA shows that one of the main organs revealing microscopic microthrombi is the pancreas (24 of 25 patients) [25]. Despite frequent pathological involvement, data on clinical expression are rare. Pancreatitis is defined as typical abdominal pain associated with a lipase level above 3 N. Although there are no solid data on its prevalence, there are several articles reporting on cases of pancreatitis in TTP. Therefore, a lipase assay should be performed at the diagnosis of TTP to detect pancreatitis [60,61,62,63,64]. Some authors even hypothesize a role for acute pancreatitis and subsequent endothelial activation in the development of TTP [65]. Indeed, true cases of TTP have been described in the follow-up of acute pancreatitis (caused by alcohol or gallstone disease), presumably due to uncontrolled systemic inflammation that may trigger the onset of TTP [66].

There are also rare reports of digestive bleeding of ischemic origin [60]. Since the introduction of caplacizumab, an increasing number of gastrointestinal bleeding episodes have been described [13,34] (Figure 1). 

### 3.5. Macrovascular Thrombosis in TTP

In a population of 55 patients admitted to the ICU for TMA, half (*n* = 28) had venous or arterial vascular thrombosis, including 7 (12.7%) patients with cerebral artery thrombosis and 21 (38%) patients (including 13 (23.6%) with central venous catheters) with deep vein thrombosis. Patients admitted to the ICU with a suspicion of TTP are treated by PEx, of which a significant proportion will be performed via a central venous line [11]. In the study by Camous et al., all patients were treated via a central venous line and the main factor associated with macrovascular thrombosis was a confirmed diagnosis of TTP [67]. This study suggests that Doppler ultrasound should be routinely performed at the site of central venous catheter insertion after its removal and as soon as there are signs of catheter dysfunction (Figure 1).

## 4. ICU Management of TTP

### 4.1. Initial Management of TTP

Patients with suspected TMA should be transferred to an ICU within a TMA referral center for rapid and specialized management. Once the diagnosis of TMA has been confirmed, the initial work-up includes the assessment of ADAMTS13 activity and ADAMTS-13 antibodies to confirm the diagnosis of TTP and eliminate differential diagnoses. Samples for ADAMTS13 activity should be taken immediately and before any PEx, but PEx should be initiated without waiting for ADAMTS13 results [68]. The initial work-up also includes an assessment of the damage of various organs that may be involved by a thorough clinical examination, biology (including cardiac troponin, lipasemia, creatininemia), a transthoracic cardiac ultrasound and cerebral imaging in the case of abnormal neurological exam [11].

The current treatment of TTP in the acute phase is based on urgent PEx, combined with corticosteroid therapy, B-cell-targeted immunotherapy, rituximab and the inhibition of the interaction between ultralarge VWF multimers and platelets, using caplacizumab, a monoclonal antibody [12].

PEx with replacement of plasma remains the cornerstone of the current management of TTP and should be started as soon as the diagnosis of TTP is suspected. The risk of death is maximal before PEx is initiated [14]. Experts recommend a daily PEx with a plasma dose of 60 mL/kg (1.5 × estimated plasma volume) until the platelet count is above 150 G/L for 48 h [68]. PEx can be administered by the peripheral venous route if available in the center, otherwise by central venous line, which can be placed with ultrasound guidance, without prior platelet transfusion.

To decrease ADAMTS-13 antibody production, adjunctive corticosteroid therapy with methylprednisolone (1 mg/kg) must be started after the first PEx and is usually given for 21 days. In the case of severe organ failure, high-dose corticosteroids (e.g., 1 g methylprednisolone) may be considered for three consecutive days [69,70]. Before the wide use of rituximab and caplacizumab, Balduini et al. compared the effectiveness of standard- versus high-dose methylprednisolone as an adjunctive treatment to PE in the acute phase of TTP. They found that high-dose corticosteroids, in association with PE, increased the rate of patients achieving complete remission [71].

Rituximab, a humanized anti-CD20 monoclonal antibody, was first introduced in patients with a suboptimal response to conventional TTP treatment. Retrospective studies showed that rituximab in association with PEx and corticosteroids resulted in fewer relapses and shorter hospitalization [72,73]. Despite the lack of a proper clinical trial in patients with severe TTP, experts recommend starting rituximab in conjunction with PEx as soon as possible [11,70]. These recommendations are supported by a meta-analysis published in 2019 of 570 patients drawn from nine studies. Patients receiving rituximab in the acute phase had a significantly lower relapse rate than those receiving conventional therapy. Similarly, the relapse rate in the rituximab group for pre-emptive treatment to prevent clinical relapse was also significantly lower than in the control group. In addition, the conventional treatment group had a significantly higher mortality rate than the rituximab group during follow-up [74].

**Figure 2:**
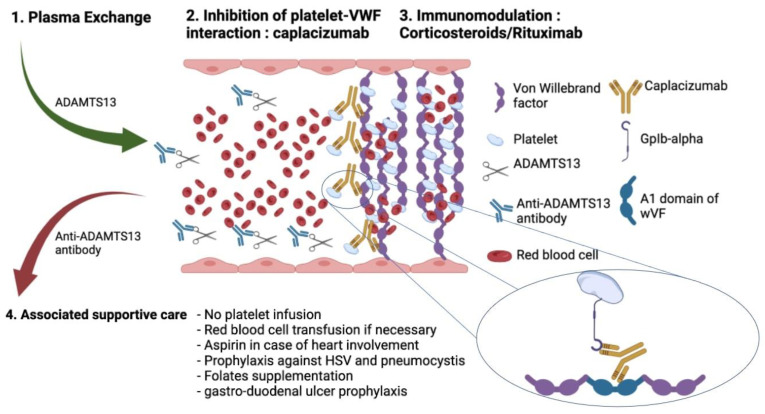
Initial management of immune thrombotic thrombocytopenic purpura (TTP). Current treatment of TTP in the acute phase is based on urgent plasma exchange (PEx), combined with corticosteroid therapy, B-cell-targeted immunotherapy, rituximab and inhibition of the interaction between ultralarge VWF multimers and platelets, using caplacizumab, a monoclonal antibody.

Caplacizumab is an anti-VWF humanized immunoglobulin targeting the A1 domain of VWF, preventing interaction with the platelet glycoprotein Ib-IX-V receptor. Prospective randomized clinical trials using caplacizumab in association with PEx, corticosteroids and rituximab in TTP showed that caplacizumab reduced platelet count time recovery, exacerbations and relapses [13,34]. Caplacizumab is administered using a 10 mg intravenous loading bolus, followed by daily 10 mg subcutaneously during PEx and for 30 days thereafter [13]. In case of severe or life-threatening bleeding, such as intra-parenchymal hematoma, caplacizumab is contraindicated (Figure 2).

### 4.2. Life-Sustaining Therapies and ICU Supportive Care

The fact that patients are immediately admitted to the ICU allows close monitoring and the rapid introduction of life-sustaining therapies. In the case of neurological injury with impaired consciousness, some patients will require mechanical ventilation to protect the upper airways. Hemodynamic failure may require norepinephrine or dobutamine in the case of severe left ventricular failure and cardiogenic shock. Emergency cardiac support using extracorporeal membrane oxygenation (ECMO) may be discussed in the case of refractory cardiogenic shock and/or refractory cardiac arrhythmia [14]. In severe AKI, RRT is readily available for patients [11].

The highest standards of ICU care must be applied, corresponding to gastric ulcer prophylaxis, the use of red blood cell transfusions if necessary, folate substitution because of acute hemolytic anemia, and deep vein thrombosis prophylaxis associated with anti-platelet therapy in patients with cardiac involvement, as soon as the platelet count is above 50 G/L [11,49,69,75]. Special attention must be paid to the prevention and surveillance of the central venous catheter for thrombosis and infection [67], maintenance of adequate blood pressure control and prophylaxis against herpes simplex virus and Pneumocystis jirovecii in the case of prolonged corticosteroids treatment [11]. Platelet transfusion should be avoided as it may aggravate microvascular damage and organ failure [76,77]. To maximize effectiveness, each drug should be given after PEx, and therapeutic drug level assessment should be performed and adjusted if necessary. Blood counts, troponin levels, hemolytic activity and ECG should be performed daily. ADAMTS13 activity and anti-ADAMTS13 inhibitors should be assessed weekly until remission [11].

## 5. Conclusions and Perspectives

Patients with TTP are at high risk of severe organ failure and ICU admission should always be considered as soon as the diagnosis of TTP is suspected. The brain, heart and kidney are the main organs that may suffer from microthrombosis. The therapeutic landscape of TTP has evolved during the last 10 years with the wider use of rituximab and caplacizumab as front-line therapy. Future ICU studies will be able to precisely determine the impact of these new standardized strategies in terms of organ failure, mortality and long-term outcomes. New promising therapies, such as recombinant ADAMTS-13, are under evaluation and may also change the future management of these patients.
